# Open data for assessing habitats degree of conservation at plot level. An example dataset of forest structural attributes in Val d'Agri (Basilicata, Southern Italy)

**DOI:** 10.1016/j.dib.2023.108986

**Published:** 2023-02-16

**Authors:** E. Carli, L. Casella, G. Miraglia, F. Pretto, I. Prisco, G. Caricato, A. Palma, P. Angelini

**Affiliations:** a Italian Institute for Environmental Protection and Research (ISPRA), Via Vitaliano Brancati, 48, Roma 00144, Italy; b Regional Agency for the Protection of the Environment of Basilicata (ARPAB), Via della Fisica 18 C/D - via della Chimica 103, Potenza 85100, Italy

**Keywords:** Habitats directive, Ecosystems assessment, Habitat monitoring, Field survey, Sustainable forests management

## Abstract

Forests supply multiple ecosystem services and host a large proportion of the Earth's terrestrial biodiversity. In particular, they provide habitats for many taxonomic groups which can be threatened by forest unsustainable management practices. Type and intensity of forest management are widely recognized as the main drivers of structure and functions in forests ecosystems. However, to better understand the impacts and the benefits deriving from forest management, there is a big need to standardize procedures of field data collection and data analysis.

Here, we provide a georeferenced dataset of vertical and horizontal structure of forest types belonging to 4 habitat types, *sensu* Council Directive 92/43/EEC. The dataset includes structural indicators commonly linked to old-growth forests in Europe, in particular the amount of standing and lying deadwood. We collected data on 32 plots (24 of 225 m^2^, and 8 of 100 m^2^, according to different forests type) during spring and summer of 2022, in Val d'Agri (Basilicata, Southern Italy). The dataset we provide follows the common national standard for field data collection in forest habitat types, published by ISPRA in 2016 with the aim to promote a greater homogeneity in assessment of habitat conservation status at Country and biogeographical level, as requested by the Habitats Directive.

**Specifications Table**

**Table:** 

Subject	Environmental Science: Management, Monitoring, Policy and Law; Nature and Landscape Conservation Agricultural science: Forestry
Specific subject area	Dendrometric measurements; Living trees, deadwood survey
Type of data	Geospatial point vector (shapefile) Table
How the data were acquired	The dataset was built by using field measurements of 32 sample plots distributed in four types of Italian zonal and azonal forests. Plots have two dimensions according to the forest type: 225 m^2^ in zonal forests, and 100 m^2^ in riparian forests. Data were acquired through the application of specific protocols, by using a free Android application (VegApp), and standardized field survey forms. The instruments we used to acquire data include meter sticks and GPS systems.
Data format	Raw
Description of data collection	Fieldwork was performed in 32 sampling sites (=plots), where we collected: *(i)* the number and the volume of living trees; *(ii)* the number of standing and downed dead trees; *(iii)* types and abundance of understory layers, included regeneration layers, such as juvenile and seedlings. We provide three datasets at the plot level, one at the tree level (xlsx format); and one shapefile at the plot level. The coordinates of each sample site are available, while the coordinates of each tree were not acquired.
Data source location	Administrative Region (NUT2): Basilicata, Val d'Agri.
Data accessibility	Repository name: Zenodo Data identification number: 10.5281/zenodo.7405293[1] Direct URL to data: https://zenodo.org/record/7405294#.Y49KgXbMKUk

## Value of the Data

• This dataset represents a monitoring baseline for the main forest habitat type in the Basilicata Region.
• If integrated with other dataset, for example the one provided by Parisi et al [2], where data for Basilicata Region are missing, the dataset can contribute to: 1) better understand the variability of the forest structural attributes in Central and Southern Italy and 2) produce the Favourable Reference Value for a list of indicators of forest ecosystems, based on real data, following the European Commission guidelines [3,4].
• The release of this dataset promotes the collaboration among researchers, monitoring and management Institutions (Administrative Regions, Regional Agencies for the Protection of the Environment).
• The active involvement of ARPA Basilicata in field data collection ensures future repeating assessments in the same localities, to evaluate the trends over time and space.
• This dataset represents a first practical example of standardization and homogenization of forest stand structure data collection for Habitat Monitoring *sensu* art. 17 of the Directive 92/43/EEC (hereafter Habitats Directive).
• Metadata will be used as a reference standard for Administrative Regions in our Country, since they derived from the National Handbook of Habitat Monitoring [5], integrated with more recent literature. Moreover, metadata will support the development of practical indications for the correct application of the monitoring techniques recommended by National Guidelines for Habitat Monitoring [5]. The main stakeholder will be the National System for the Protection of the Environment (SNPA), but all the bodies involved in environmental monitoring activities will also benefit.

## 1 Objective

With the release of the dataset, ISPRA, in collaboration with ARPA Basilicata, decided to practically test on the field how Administrative Regions, which are responsible for habitat monitoring activities, can collect data on forests structure easily but efficiently. At the end of the project, ISPRA will produce practical indications for the correct application of the monitoring techniques recommended by the National Handbook of Habitat Monitoring [5].

## 2 Data Description

Table 1 shows the characteristics of the sample sites, and information on type of management, when available, as follows.

**Table 1:** Sample site description.

Variable	Description	Measure unit
ID_plot	Identification number of the plot	-
Data	Data	DDMMYYYY
X	Coordinate of the plot centre (longitude)	EPSG: 4326
Y	Coordinate of the plot centre (latitude)	EPSG: 4326
Z	Altitude of the plot centre	m a.s.l.
Aspect	Mean aspect of the plot	-
Slope	Mean slope of the plot	%
Area	Plot area (which can be 100 or 225 m^2^)	m^2^
Shape	Plot shape (rectangular or square)	-
Rocks	Cover of rocks	%
Stones	Cover of stones	%
FMP	Forest Management Plan in force	-
Mng_type	Management type (unmanaged, high forest, coppice, NA)	-
Veg_type	Vegetation type (riparian forest, oak forest, beech forest)	-
T_species	Dominant tree species	-
Stage	Successional stage (neoformation, young, mature, old-growth forest)	-
Repr_type	Vegetative or sexual reproduction (veg/seeds/veg+seeds)	-
HT	Habitat type code (Annex 1 of Habitats Directive)	-

The same information included in Table 1 are also reported in the shapefile that locates the sample sites.

Table 2 provides the description and the measuring unit of all the attributes included in the dataset at plot level. It includes parameters used for assessing old-growth forests [6], [7], [8], linking them to the main type forests management, in order to both select indicators useful for assessing the conservation degree of forest habitat types, and identify good practices for nature conservation in these ecosystems.

**Table 2:** Forest site attributes.

Variable	Description	Measure unit
ID_plot	Identification number of the plots	-
Mun	Municipality in which the plot occurs	-
C_veg_tot	Total vegetation cover	%
C_bare	Bare ground cover	%
C_soil	Soil cover	%
C_litter1	Litter cover (< 2 cm depth)	%
C_litter2	Litter cover (> 2 cm depth)	%
C_T1	Tree high layer cover	%
C_T2	Tree low layer cover	%
C_SH1	Shrub high layer cover	%
C_SH2	Shrub low layer cover	%
C_herb	Herb layer cover	%
C_J	Juvenile layer cover	%
C_S	Seedlings layers cover	%
H_T1_max	Max tree high layer height	m
H_T1_avg	Mean tree high layer height	m
H_T2_max	Max tree low layer height	m
H_T2_avg	Mean tree low layer height	m
H_SH1_max	Max shrub high layer height	m
H_SH1_avg	Mean shrub high layer height	m
H_SH2_max	Max shrub low layer height	m
H_SH2_avg	Mean shrub low layer height	m
H_herb_max	Max herb layer height	m
H_herb_avg	Mean herb ayer height	m
Epyphytes	Epiphytes occurrence	(present, abundant, absent)
Non_vasc	Lichens and mosses occurrence	(present, abundant, absent)

Table 2 contains information on structure at plot level.

Table 3 contains information on living trees and deadwood at plot level.

**Table 3:** Living trees and deadwood at plot level.

Variable	Description	Measure unit
ID_plot	Identification number of the plots (the same of Table 1)	-
T_num	Number of living trees exceeding 10 cm DBH per plot	-
BA_T	Basal area of living trees exceeding 10 cm DBH	m^2^
V_T	Tree volume per plot BA × average height of tree layers	m^3^
N_SDT	Number of standing dead trees per plot	-
n_DDT	Number of downed dead trees per plot	-
N_stumps	Number of stumps per plot	-
N_snags	Number of snags per plot	-
N_old_T	Number of old trees per plot	-
N_MT	Number of trees with cavities (as surrogate of microhabitat) per plot	-

Table 4 describes the dataset related to single-tree data, and it contains the following fields for all living trees, exceeding 10 cm DBH, occurring in the plots. The nomenclature follows [9,10]:

**Table 4:** Data collected at single-tree level.

Variable	Description	Measure unit
ID_Plot	Identification number of the plots (the same of Table 1)	-
ID_tree	Identification number of the tree	-
Species	Tree species	-
DBH	Diameter at breast high	m
BA_T	Basal area of the tree	m^2^

## 3 Experimental Design, Materials and Methods

Dataset refers to the upper and middle valley of the Agri river, one of the main valleys of the Basilicata Region, located along the main axis of the Southern Apennines. The Agri river starts from Serra di Calvello, at 1567 m a.s.l., and flows between the Mount Calvelluzzo (1699 m), Mount Volturino (1835 m), and Mount of Viggiano (1727 m) on the left bank, and Maddalena Mountains (between 1300-1500 m a.s.l.) on the right bank, which represent the natural border between Campania and Basilicata. The investigated area, spreading for 260 km^2^ and including some high ground, as Mount of Viggiano, Aquila Mount (1096 m a.s.l.) and Mount Calvarosa (1261 m a.s.l.), ends when the Agri river reaches the Pertusillo Lake (532 m a.s.l.), an artificial reservoir that dates 1957-1962.

Along the slopes, area mainly hosts *Quercus cerris* dominated forests, which is the most spread woods in Basilicata Region; in the South-Eastern part of the area, close to the Pertusillo Lake, the main ecosystems are represented by mixed oak forests (*Quercus cerris* and *Quercus frainetto*, belonging to the habitat 91M0); *Q. pubescens* is relatively rare in the area and it is not associated to any habitat type. *Fagus sylvatica* forests (habitat 9210*) can only be found in Viggiano Mountain and Aquila Mountain. Among the azonal forests, two riverine woods can be identified, the first one dominated by *Populus nigra* and/or *Salix alba* (habitat 92A0), the second one characterized by *Alnus glutinosa* (belonging to the habitat 91E0) [11,12]. Furthermore, the area hosts one of the main centers of Oil exploitation in Basilicata and in Italy.

**Fig 1:**
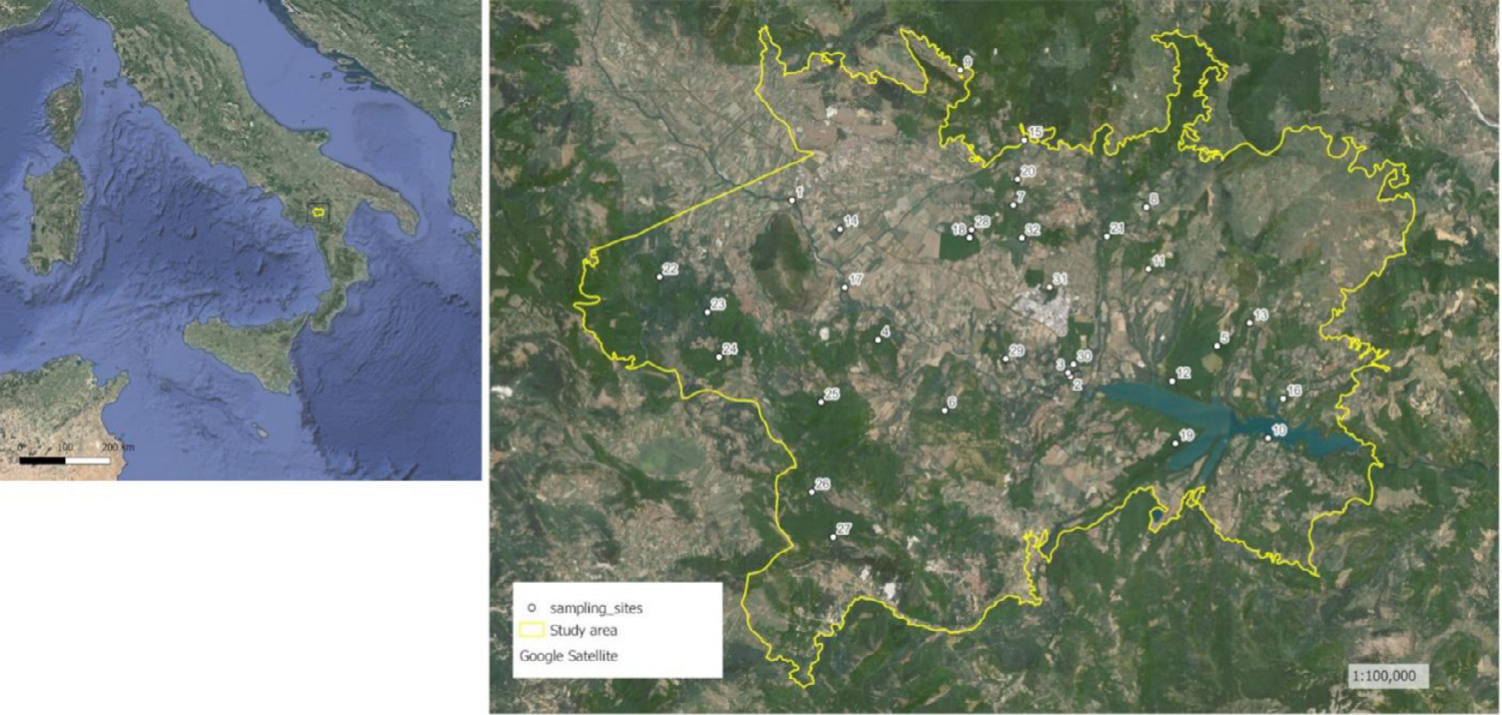
The investigated area and the location of sampling sites.

Fig. 1 shows the investigated area and the location of sample sites.

We used a random-stratified sampling design according to the different forest ecosystem types recognized in the Map of the Nature, a national project for land management and environmental protection produced by ISPRA for each administrative region. The Basilicata Map of the Nature was released in 2012 at a scale of 1:50.000 [13], and it includes a total of 86 habitat types and, in particular, 23 forest habitat types. Each landscape unit (polygon) in the Map of the Nature is also associated to an assessment of the “ecological value” [14], defined as a “naturalistic quality”, calculated through the use of specific indicators and applied at regional level [15]. We used the attribute “ecological value” as a proxy of the human impact that occurs in each forest polygon within the study area.

The selection of the location of the sample sites were spatially balanced by using the software QGis vers. 3.22 [16]. Environmental data of the sample sites (=plots) are shown in Table 1.

Sample sites were located in the field by using GPS in order to identify the centre of the plot. Plots have square shape (225 m^2^) in zonal forests (*Quercus* or *Fagus* dominated forests); and rectangular shape (100 m^2^ i.e., 20 m × 5 m) in azonal riparian forests (*Salix, Populus* or *Alnus* dominated forests). Shapes and dimensions, follow the standards of National and European Handbook for habitat monitoring activities [5,17].

In each plot, we recorded, by visual assessment, the percentage cover of deadwood, and percentage cover of living trees. Particularly, we collected the percentage cover of each vegetation layer (included juvenile and seedlings, which are in general neglected compared to the other layers but are indeed important as surrogate of the resilience of the forests), the highest and average heights of trees, shrubs, and herbs layers. We also measured the DBH of living trees at individual level by using meter sticks. Finally, we recorded the number of standing dead trees, downed dead trees, stumps, snags, old trees, and trees with cavities per plot (Table 3).

Visual estimates are often applied in vegetation cover studies as they are relatively rapid and well established, although potentially less robust than other methods; to reduce the degree of subjectivity data were collected by a unit of only 4 researchers in a very short period (from May to July 2022).

For future monitoring activities, we suggest the use of innovative technologies such as laser scanner, in order to provide more accurate measurements for the assessment at the national spatial scale.

Concerning data collection, we use standardized field survey forms, and “VegApp”, a free application on Android platform (https://vegapp.de/), which allows to collect structural and vegetation data on the field, storing them directly in a georeferenced database.

Lastly, in order to stress the importance of forests management plans to provide useful data for the assessment of conservation status of the habitat types [18], we recorded the type of management, according to forestry management plans available for two of the involved Municipalities, Viggiano and Grumento Nova, (http://valutazioneambientale.regione.basilicata.it/valutazioneambie/detail.jsp?sec=104357&otype=1011&id=104976, http://valutazioneambientale.regione.basilicata.it/valutazioneambie/detail.jsp?sec=102944&otype=1011&id=103612; accessed 05/12/2022). Forestry management plans still lack for some Municipalities. In this case, we indicated the type of management according to field survey observations.

## Ethics Statements

The authors declare that the present work did not include experiments on human subjects and/or animals.

## CRediT authorship contribution statement

**E. Carli:** Methodology, Writing – original draft, Investigation, Writing – review & editing. **L. Casella:** Conceptualization, Supervision, Writing – review & editing. **G. Miraglia:** Investigation, Data curation, Writing – review & editing. **F. Pretto:** Writing – original draft, Investigation, Data curation, Writing – review & editing. **I. Prisco:** Investigation, Writing – review & editing. **G. Caricato:** Conceptualization, Writing – review & editing. **A. Palma:** Conceptualization, Writing – review & editing. **P. Angelini:** Conceptualization, Supervision, Writing – review & editing.

## Declaration of Competing Interest

The authors declare that they have no known competing financial interests or personal relationships that could have appeared to influence the work reported in this paper.

## Data Availability

Open data for assessing habitats degree of conservation at plot level. An example dataset of forest structural attributes in Val d'Agri (Basilicata, Southern Italy) (Original data) (Zenodo).
